# An Insight into Storage Lipid Synthesis by *Yarrowia lipolytica* Yeast Relating to Lipid and Sugar Substrates Metabolism

**DOI:** 10.3390/biom9110685

**Published:** 2019-11-01

**Authors:** Agata Fabiszewska, Paulina Misiukiewicz-Stępień, Magdalena Paplińska-Goryca, Bartłomiej Zieniuk, Ewa Białecka-Florjańczyk

**Affiliations:** 1 Department of Chemistry, Faculty of Food Sciences, Warsaw University of Life Sciences, 02-776 Warsaw, Poland; bartlomiej_zieniuk@sggw.pl (B.Z.); ewa_bialecka_florjanczyk@sggw.pl (E.B.-F.); 2 Postgraduate School of Molecular Medicine, Medical University of Warsaw, 02-091 Warsaw, Poland; paulina.misiukiewicz@gmail.com; 3 Department of Internal Medicine, Pulmonary Diseases and Allergy, Medical University of Warsaw, 02-097 Warsaw, Poland; mpaplinska@wum.edu.pl

**Keywords:** *ACL* gene, acyl-CoA oxidase, ATP-citrate lyase, *POX2* gene, single cell oil, *Yarrowia lipolytica*

## Abstract

Single cell oil (SCO) is the lipid accumulated in the cells of oleaginous microorganisms. Cellular lipids can be synthesized in two different pathways: de novo by metabolizing hydrophilic substrates and ex novo by fermenting hydrophobic substrates. The aim of the study was to evaluate the effect of carbon source (glucose and olive oil) in the culture medium on the course of microbial oil accumulation in *Y. lipolytica* cells. The level of selected gene expression by real time quantitative PCR method was investigated. The significant increase in expression of the *POX2* gene encoding acyl-CoA oxidase II, which preferentially oxidizes long-chain acyl-CoAs formed from substrate fatty acids incorporated inside the microbial cell, was observed in medium with olive oil in relation to glucose containing medium. Noteworthily, the presence of lipid carbon substrate did not inhibit the level of *ACL* gene transcription coding for ATP-citrate lyase, the key enzyme of the lipid de novo accumulation process. The present study indicated that de novo lipid biosynthesis could occur despite the presence of fatty acids in the medium, and the synthesis of storage lipids in the presence of lipid carbon substrates could be carried out with the use of both pathways (de novo and ex novo).

## 1. Introduction

*Yarrowia lipolytica* is a model species of oleaginous yeast. The fully sequenced genome and well-known metabolism of the yeast makes *Y. lipolytica* currently a model species in much basic research in the fields of protein secretion and peroxisome biogenesis, and research on the complex I of the respiratory chain and the metabolism of hydrophobic substrates [1,2]. Efficient methods for the synthesis of enzymes e.g., lipases and organic acids such as citric acid in *Y. lipolytica* culture, have been developed on a laboratory scale. The yeast species was also developed as an erythritol producer (a sweetener used as a substitute for sucrose), but currently the most attention is paid to single cell oil (SCO) biosynthesis [3].

SCO is a microbial oil produced by oleaginous microorganisms, which can accumulate lipids to more than 20% *w*/*w* in their dry cell. The microbial oil isolated from yeasts consists mainly of triacylglycerol molecules containing unsaturated fatty acid residues. The storage of lipids in oleaginous yeast proceeds with the specific incorporation of aliphatic lipophilic residues of acylglycerols and the intracellular transformation of these substrates [4,5]. The modified lipids are stored in lipid bodies. SCO has industrial interest due to its particular and precise biochemical and physicochemical properties [6]. Of significant importance is the utilization of yeast lipids as substitutes of high added value exotic fats, e.g., cocoa butter and common vegetable oils, and finally, SCO can be produced as a starting material of 2nd generation biodiesel [7].

Oleaginous yeast synthesize lipids by one of two different biochemical pathways depending on the carbon substrate used in the culture medium. De novo lipid accumulation involves formation of acetyl-CoA resulting from the inhibition of the Krebs cycle in sugar-based media. The ex novo route is characterized by incorporation of final products or intermediates of fatty acid β-oxidation into triacylglycerol molecules in media containing hydrophobic carbon sources [8].

The first step of lipid metabolism has been studied in enzymatic and molecular level in significant detail by non-conventional oleaginous *Y. lipolytica* yeast. The *LIP* family genes encoding lipases and *POX* family genes coding for acyl-CoA oxidases are characterized in detail in transcriptional and translational levels. Meanwhile, the further steps of ex novo fatty acid synthesis in media with hydrophobic substrates are less widely described. Most reports on this issue came from the late 1990s and the beginning of the 21st century. The biochemistry of lipid accumulation in oleaginous microorganisms was described by observations of species other than *Y. lipolytica*, such as *Candida curvata, Cryptococcus curvatus, C. albidus, Cunninghamella echinulata, Mortierella isabellina, M. alpina, Mucor circinelloides, Lipomyces starkeyi*, and *Rhodosporidium toruloides* [5,9,10]. There exists a common knowledge on incompatibility of the presence of hydrophobic substrates in culture medium with de novo biogenesis of cellular lipids. The thesis is supported by the inhibition of aliphatic exogenous chains of two crucial de novo route enzymes: fatty acid synthetase and ATP-citrate lyase [5].

The paper attempts to evaluate the effect of carbon source (glucose and olive oil) in the culture medium on the course of microbial oil accumulation in *Y. lipolytica* cells. Two genes are taken into consideration: *POX2* and *ACL* genes. The *POX2* gene encodes acyl-CoA oxidase II, which preferentially oxidizes long-chain acyl-CoAs formed from substrate fatty acids incorporated inside microbial cells, which is the first step in the ex novo route [11]. The *ACL* gene codes for ATP-citrate lyase, the key enzyme of the lipid de novo accumulation process [8]. Composition of fatty acids in storage lipids and kinetic parameters of batch bioreactor cultures taken from phenotypic observations were correlated with the level of selected gene expression by real time quantitative PCR method (real time-qPCR) to reveal the possibility of performing de novo biogenesis of cellular lipids in spite of the presence of an exogenous aliphatic substrate in culture medium and the absence of glucose.

## 2. Materials and Methods 

### 2.1. Microorganism and Culture Conditions

*Y. lipolytica*, strain KKP 379 from the Collection of Industrial Microorganisms at the Institute of Agricultural and Food Biotechnology in Warsaw, was used. The strain was stored in cryovials containing ceramic beads with a cryopreservative fluid (Protect Select, Technical Service Consultants Ltd., Heywood, United Kingdom) at −20 °C. There were three different culture media used: YPG, MO5, and MG7. YPG medium contained (g/L): glucose, 20; peptone, 20; and yeast extract, 10 (BTL, Łódź, Poland). MO5 and MG7 media contained (g/L): KH_2_PO_4_, 7; (NH_4_)_2_SO_4_, 2.5; Na_2_HPO_4_, 2.5; FeSO_4_ × H_2_O, 0.16; CaCl_2_, 0.15; MnCl_2_ × 4H_2_O, 0.08; yeast extract, 2.0; and peptone, 1.0. All inorganic salts were purchased from Avantor Performance Materials Poland S.A. (Gliwice Poland). The carbon sources used in those media were: glucose in MG7 medium (70 g/L, BTL, Łódź, Poland) and olive oil (50 g/L, Aceites Borges Pont, Terrega Spain). MO5 medium contained 1 g/L of Tween 80 (Sigma Aldrich, Saint Louis, MO, USA). The initial pH of medium was estimated at 6.0.

The inoculum culture was performed in 100 cm^3^ YPG medium and incubated for 20 h at 28 °C in an IKA KS 4000 ic control shaker (IKA company, Konigswinter, Germany) at 150 rpm. Batch cultures were carried out in a BIO FLO 3000 bioreactor (New Brunswick, Hamburg, Germany) at 28 °C, 350 rpm agitator speed, and 0.025% (*v*/*v*) inoculum. Medium was aerated with compressed air at a flow of 105 L/h per 1 L medium. Inoculum for the bioreactor was standardized by measuring the optical density of the culture.

### 2.2. Determination of Biomass Yield, Glucose Concentration, pH, and Oxygen Consumption during Batch Culture

Changes in the growth phases of *Y. lipolytica* were evaluated on the basis of changes in the level of medium oxygenation, and dissolved oxygen was measured by means of an oxygen electrode. The oxygen consumption was the parameter characterizing dissolved oxygen concentration in relation to its content at the beginning of yeast culture. To determine the pH of cultures, a pH glass electrode was used. Biomass yield was evaluated by the thermogravimetric method, and cells were harvested by centrifugation (8000 rpm, 10 min, 4 °C), washed in distilled water, and dried at 105 °C until constant weight. Glucose concentration in medium was determined by the 3,5-dinitrosalicylic acid (DNS) colorimetric method at 540 nm [12]. Kinetic parameters of storage lipid biosynthesis in a batch culture of *Y. lipolytica* were calculated according to Papanikolaou and Aggelis [7].

### 2.3. Lipid Extraction and Analysis

Cellular lipids were extracted according to the method described previously [13]. Biomass for lipid extraction was harvested by centrifugation (8000 rpm, 10 min, 4 °C), washed with distilled water, and dried at 80 °C. Extraction was performed in a Soxhlet extractor using *n*-hexane as a solvent. Olive oil concentration in medium after the yeast culture was determined by twice lipid extraction from 200 cm^3^ of medium with *n*-hexane. The weight of extracted oil was referenced to the volume of culture medium or to the cell dry mass of the yeast biomass sample. Fatty acid composition in cell lipids was evaluated by gas chromatography. Fatty acid derivatization was provided using 1 M sodium methoxide and 10% BF_3_. FAMEs (fatty acids methyl esters) were extracted with 4 mL of *n*-hexane. The hexane phase was analyzed with GC–FID. Methyl heptadecanoate was used as an internal standard. A gas chromatograph 68790 N (Agilent Technologies, Foster City, CA, USA) equipped with a capillary column HP 5-MS was used for the analysis (column parameters: 0.25 mm internal diameter and 0.25 μm stationary phase film thickness). Helium was used as the carrier gas. Individual fatty acids were identified on the basis of retention times, comparing them with reference ones.

### 2.4. Determination of the mRNA Transcription Profiles of POX2 and YALI0 Genes

#### 2.4.1. RNA Isolation 

Cells destined for extraction of RNA were preserved in RNAlater reagent (Thermo Fisher, Waltham, MA, USA) and kept at −80 °C. Samples were refrigerated at room temperature for 15 min and centrifuged at 10,000× *g* for 5 min. Extraction of RNA was performed by Chomczynski and Sacchi’s [14] methodology. Biomass was lysed in Trizol (Thermo Fisher, Waltham, MA, USA) except for biomass from the culture medium that contained olive oil, which was lysed with QIAzol Lysis Reagent (Qiagen, Hilden, Germany) for 15 min, then crushed in a hand held homogenizer. Then 200 μL of chloroform (Sigma Aldrich, Saint Louis, MO, USA) was added to the powder and dynamically shaken manually for 15 s. After 10 min of incubation in room temperature, samples were centrifuged at 12,000× *g* at 4 °C for 15 min. The water phase was transferred to a new tube containing 500 μL isopropyl alcohol (Sigma Aldrich, Saint Louis, MO, USA) and centrifuged. The pellet was suspended in 1 cm^3^ of 75% ethanol (Sigma Aldrich, Saint Louis, MO, USA) and centrifuged at 12,000× *g* at 4 °C for 5 min. The pellet was dried on ice. RNA was suspended in 30 μL of sterile water, and absorbance was measured at 260 and 280 nm. The integrity of isolated RNA was checked by electrophoresis in a 1% agarose gel. Green Stain Fluorescent Nucleic Acid (Cyanagen, Bologna, Italy) was used for identification of the rRNA subunits.

#### 2.4.2. Reverse Transcription and Real Time PCR

A Maxima First Strand reagent cDNA Synthesis Kit for real time-qPCR with dsDNase (Thermo Fisher, Waltham, MA, USA) was used. The reaction mixture (20 μL) containing 1 μg RNA in 8 μL volume, 1 μL reagent 10× dsDNAase buffer, and 1 μL dsDNAse were incubated for 2 min at 37 °C and for 1 min at 4 °C. In the second stage, 4 μL of the 5× Reaction Mix, 2 μL of Maxima enzyme, and 4 μL of water were added to 10 μL of the reaction mixture from the first stage. The PCR conditions were: 10 min at 25 °C; 20 min at 50 °C; and 5 min at 85 °C. The cDNA was stored at −80 °C.

Real time quantitative PCR (real time-qPCR). All real time-qPCR reactions were carried out in Prism 7500 (Applied Biosystems, Foster City, CA, USA) in a volume of 16 μL. The reaction mixture consisted of 8 μL TaqMan master mix, 0.48 μL of each primer (150 nM), 0.4 μL of probe (100 nM), 5.64 μL sterile water, and 1 μL cDNA. The PCR conditions were: 50 °C for 2 min, then 95 °C for 10 min, followed by 40 cycles of 95 °C for 15 s, and 60 °C for 1 min.

Sequences for primers and probes:

*POX2*

FW: GAT GGA TCC AGT TCA CCA ACG T

REV: GTT ACC CTC TCG GTC GAC CTT T

PROBE: CCC CCG ACA GAA CCT GCT CAT GAA

*ACL*

FW: TTC CGA ACC ATC GCC ATT A

REV: TGG GCC TTG TGG AGG ATC T

PROBE: CCC GAG CGA CGA GCC CGA

18s rRNA: Eukaryotic 18s rRNA Endogenous Control (Hs99999901_s1)

The nucleotide sequences were identified using the BLAST search at NCBI.

The constitutive gene—18s rRNA served as an internal control gene to normalize the level of *POX2* and *ACL* gene expression. The results were expressed in relative quantification units as a relative expression of the gene tested in relation to the constitutive gene. CT (threshold cycle number in which the fluorescence of a given sample exceeds a threshold value) for a given amplicon and CT for endogenous control (18s rRNA) were determined for each sample. By calculating ^Δ^CT, i.e., the difference in CT of the test and the CT gene, the gene expression level for the constant volume of the nucleic acid was normalized. From ^Δ^CT of each biomass sample grown on a medium with glucose or oil, ^Δ^CT of biomass grown on a standard substrate (control) was also subtracted, resulting in ^ΔΔ^CT. The level of changes in the expression of genes tested by normalized endogenous control was calculated automatically from the ^2-ΔΔ^CT formula [15].

### 2.5. Statistical Analysis

The results obtained in the CT values from real time-qPCR experiments were analyzed using a U Mann-Whitney test (Statistica 9.0, Statsoft, Kraków, Poland). Statistical analyses of results on fatty acid content were performed of repeated measurements with one-way ANOVA followed by Tukey’s multiple comparison test and analysis of correlation using STATISTICA 13.0 (Statsoft, Kraków, Poland). *p* values of *p* ≤ 0.05 were considered to be statistically significant.

## 3. Results

### 3.1. Impact of Carbon Source on Synthesis of Lipids in Yeast Cells

**Figure 1:**
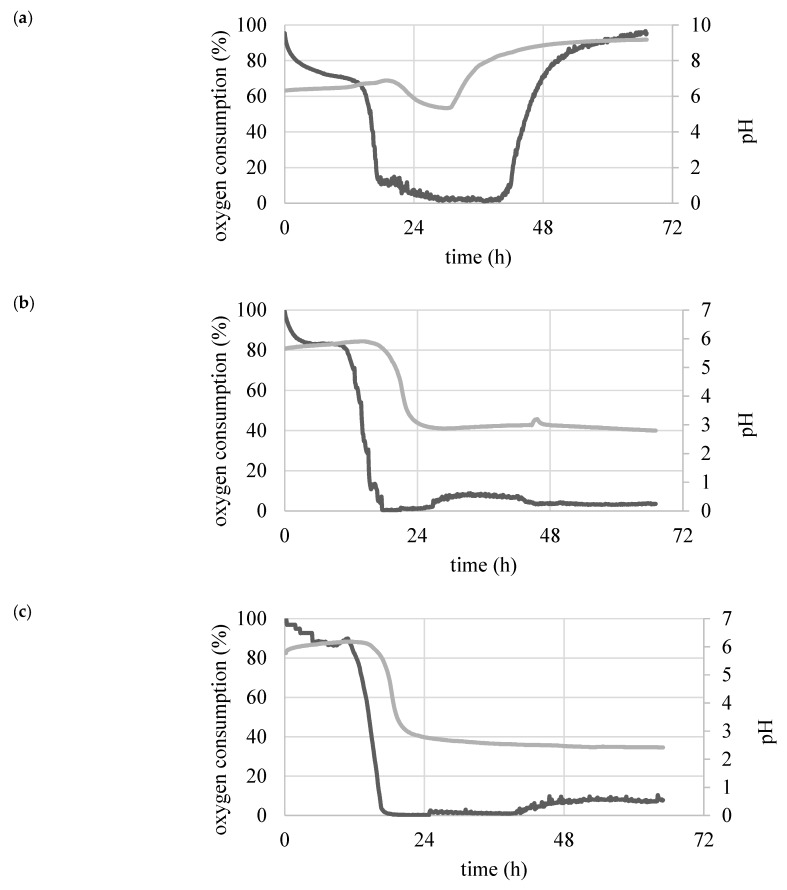
Changes in pH (light line) and oxygen consumption (dark line) during a batch culture of *Y. lipolytica* KKP 379 strain in media: (**a**) YPG, (**b**) MG7, (**c**) MO5.

Yeast were grown in control medium (YPG) and two experimental media (MO5 and MG7). pH and oxygen consumption were constantly determined (Figure 1) as they corresponded with the phases of cell growth. The lag phase lasted for about 12 h in MO5 and MG7 media, followed by an exponential phase, which lasted until the end of the experiment. The literature data showed that the by-product obtained both during the synthesis of microbial lipids in medium containing glucose (de novo route) and lipids (ex novo route) were organic acids, including citric acid [16]. These compounds were mainly responsible for significant reduction in the pH of medium in both cultures.

In the YPG control medium there was a significant decrease in the degree of oxygenation of the medium after 15 h, when cells entered the log growth phase, which lasted over a day. The pH of the YPG medium remained high throughout the entire duration of the culture. During cultivation in YPG control medium, SCO was not produced and a conversion yield of storage lipids per biomass was 0.004 g/g d.w. (dry weight) at 65 h. This was due to the composition of the medium used, which contained a significant amount of nitrogen source. The control medium used was characterized by a high nitrogen content, i.e., a small C:N ratio, whose high value is indispensable for the synthesis of de novo storage lipids and synthesis of by-products such as organic acids [3].

Storage lipid production was not significantly different between cultures where glucose or olive oil were used as carbon substrates at the end of the experiment (Table 1), but a relevant high content of storage lipids was obtained in the early lag phase in MO5 medium (23 h) when it amounted to 0.207 g/g d.w. The synthesis of cellular lipids proceeded intensely during the first day in medium with olive oil as opposed to yeast culture in medium with glucose and control medium, where the analogous process took place in the late logarithmic growth phase. The highest value of the maximum concentration of lipid coefficient (L), which is one of the most important parameters describing the kinetics of microbial lipid synthesis, was observed also for olive oil medium, where it amounted to 1.468 g/L in 23 h. Concentration of lipids in MG7 medium was lower by almost 30%, and in the control medium the value accounted for less than 4% of the L parameter calculated for the MO5 medium.

### 3.2. Fatty Acid Composition in Microbial Oil from Y. lipolytica

**Figure 2:**
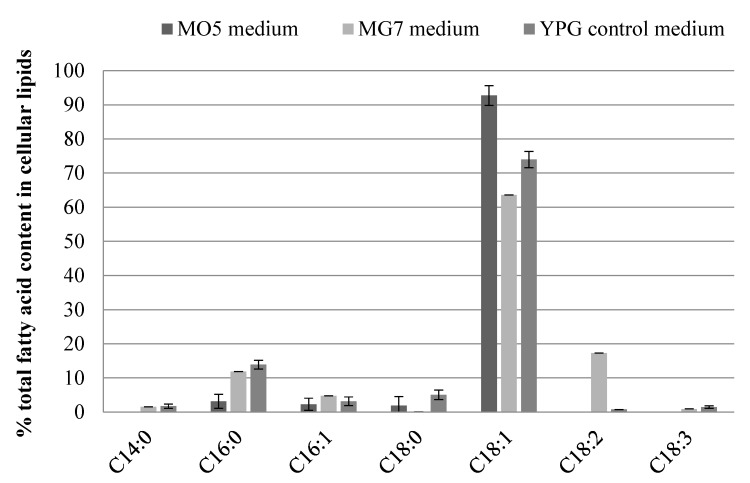
The composition of fatty acids in lipids synthesized in cells during *Y. lipolytica* batch cultures in MG7 (containing glucose), MO5 (containing olive oil), and YPG control media (abbreviations: C14:0-myristic acid, C16:0-palmitic acid, C16:1-palmitoleic acid, C18:0-stearic acid, C18:1-oleic acid, C18:2-linoleic acid, C18:3-linolenic acid).

To compare the changes in the composition of fatty acids in cellular lipids depending on the carbon source in medium, samples from the control medium and from experimental media were analyzed (Figure 2). Monounsaturated oleic acid (C18:1) was found in the highest concentration in each microbial oil independently of growth medium in which yeast were cultured, respectively 92.72% for MO5, 73.96% for YPG, and 63.56% for MG7. The presence of saturated acids, palmitic acid in the amount of 3.14% and stearic acid (C18:0) in the amount of 1.87%, was also found in the microbiological oil from yeast grown in MO5 medium, as well as monounsaturated palmitooleic acid (2.28%). In the sample derived from the MG7 medium, the amount of linoleic acid, which was 17.29%, was also significant. The palmitic acid content in MG7 medium and control medium was similar—11.87% and 13.91%, respectively. The acids differentiating the oils were myristic acid (14:0), linoleic acid (18:2), and linolenic acid (18:3), which were not detected in oil extracted from cells cultured in medium with olive oil.

**Figure 3:**
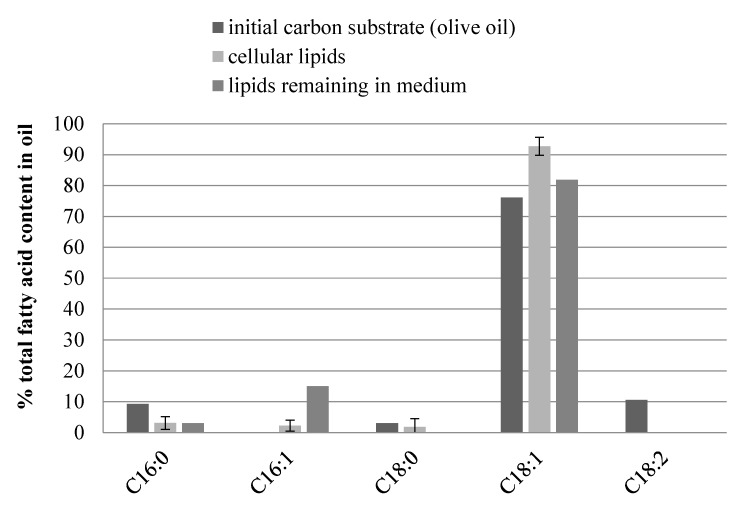
Comparison of fatty acid content in lipids synthesized in cells during *Y. lipolytica* batch cultures in MO5 medium with fatty acid content in initial carbon substrate and lipids remaining in culture medium–residual oil (abbreviations: C16:0-palmitic acid, C16:1-palmitoleic acid, C18:0-stearic acid, C18:1-oleic acid, C18:2-linoleic acid).

An interesting relation could be observed when analyzing changes in the composition of fatty acids present in acylglycerols in cellular lipids and in olive oil constituting the initial carbon source in medium and the composition of fatty acids in the oil remaining in the medium after yeast growth (Figure 3). Olive oil used as a carbon substrate contained the highest content of oleic acid (76.10% of all fatty acids) in comparison to the oil remaining in the medium (84%, Figure 3). In the initial carbon source, palmitic (C16:0) and linoleic (C18:2) acids were detected in the amount of 9.30% and 10.60%, respectively. No palmitoleic acid (16:1) was found in the olive oil as opposed to the residual oil, where it accounted for 15.06%. On the other hand, no stearic acid was detected in lipids remaining in culture medium. The residual oil not used by *Y. lipolytica* yeast growth was therefore less diverse in terms of the fatty acid composition than the initial carbon substrate.

Cellular lipids showed higher levels of oleic acid than the initial carbon source (92.72% and 76.10% respectively). This fact could be related to the selective uptake of this acid by yeast cells and its accumulation inside microorganisms by the ex novo route. In this case, the differentiating acids were also palmitoleic acid, present only in microbial oil in the amount of 2.28%, and linoleic acid found in the olive oil in amount of 10.60%.

### 3.3. Transcription Level of Genes Involved in De Novo and Ex Novo Storage Lipid Synthesis

**Figure 4:**
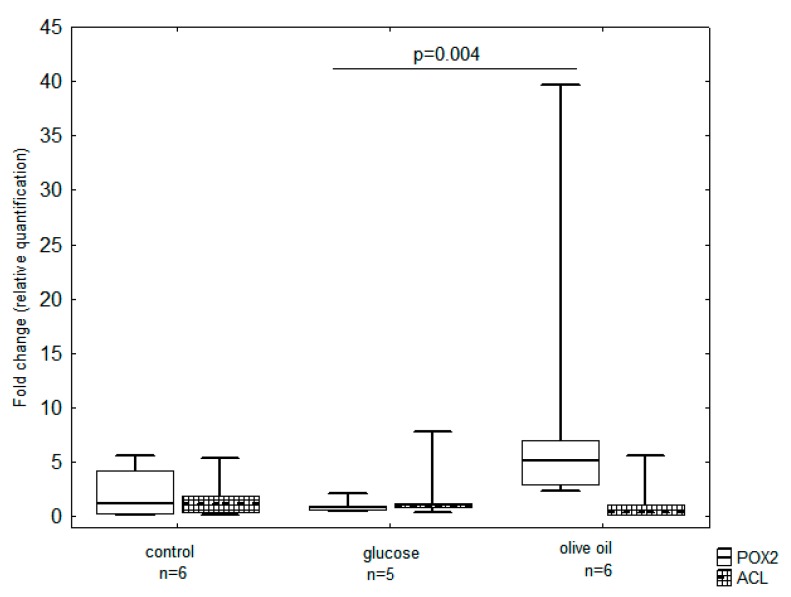
Average expression of the *POX2* and *ACL* genes in yeast cells of *Y. lipolytica* KKP 379 during batch cultures in control YPG medium, MG7(containing glucose), and MO5 (containing olive oil) media. The results are presented as the median (line), the percentile range (frame), and the range of minimum-maximum.

The total RNA was isolated from yeast cells grown in media differing in the carbon source (glucose or olive oil), and the level of changes in *POX2* and *ACL* gene expression is presented in Figure 4. The *POX2* and *ACL* gene expression in the control group, which was the yeast biomass grown in YPG medium, was 1.24 and 1.22, respectively. In the biomass of yeast grown in MG7 medium, both gene transcriptions were statistically insignificant (0.86 and 1.0 respectively). A different level of transcription of the studied genes was noted in the case of yeast biomass grown in MO5 medium. The level of *POX2* gene expression was 5.16 and was significantly higher than cells grown in control and MG7 media.

**Figure 5:**
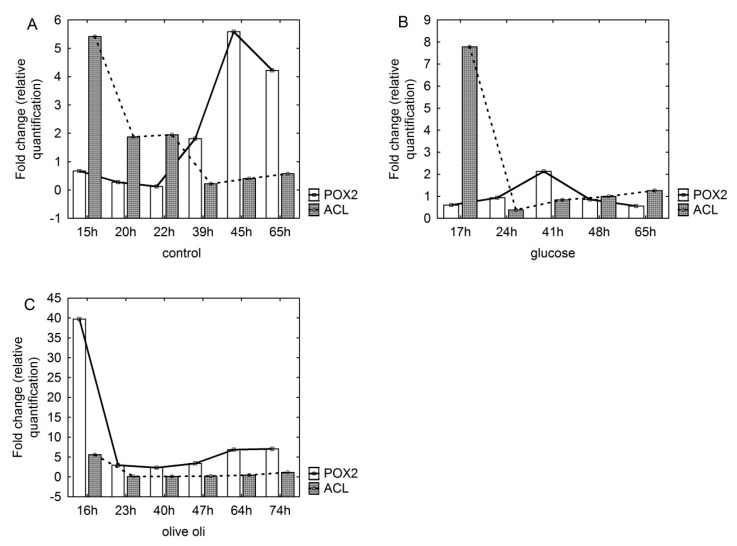
Changes in *POX2* and *ACL* gene expression during *Y. lipolytica* batch bioreactor culture in (**A**) control YPG medium, (**B**) MG7 medium (containing glucose) and (**C**) MO5 medium (containing olive oil).

The changes in *POX2* and *ACL* gene expression were also analyzed over time in batch bioreactor culture. The expression profile of genes for cells sampled from YPG control medium showed a much larger variation as reflected in their Ct values (Figure 5A). It should be outlined that the content of glucose in the 22nd hour of the experiment was only 6 g/L and at 39th h the substrate was almost completely consumed by the yeast (1 g/L, data not shown on the figure). The highest consumption of carbon substrate was in the logarithmic growth phase, and the most noticeable decrease in the substrate content in experimental media was during the first day of culture (from 50 g/L to 23 g/L for MG7 medium and from 70 g/L to 37 g/L for MO5 medium, respectively). During the first day of growth, *ACL* gene expression was significantly higher compared to *POX2* growth when storage lipid synthesis occurred simultaneously with yeast growth, while when entering the stationary growth phase, the level of expression of the gene began to decrease with simultaneous increases in the level of *POX2* gene expression. Such a state could be caused by the exhaustion of the carbon source in the medium and the beginning of metabolism of stored intracellular lipids by β-oxidation [17]. The *ACL* gene in yeast cells grown in MG7 medium was found to be expressed similarly to the profile in YPG medium, exhibiting the highest expression at the beginning of the lag phase growth (Figure 5B). There was no increase in the level of expression of the gene during the log phase, and the expression of the *POX2* gene throughout the culture was low and statistically insignificant. Interestingly, at the initial exponential growth phase of yeast cells sampled from MO5 medium, significantly high *POX2* gene expression was observed, which decreased over time (Figure 5C).

## 4. Discussion

### 4.1. Single Cell Oil Yield and its Quality in Dependence with Carbon Substrate

*Y. lipolytica* shows the ability to use lipids as a source of carbon, therefore reducing the content of storage lipids during the exponential phase could be caused by taking a certain amount of lipids from lipid bodies as a substrate in β-oxidation [17]. The molar C:N ratio in both experimental media (MO5 and MG7) was slightly different: 60:1 for MG7 medium and 85:1 for the MO5 medium. Extending the duration of the log phase in MO5 medium increased only the conversion yield of biomass per carbon substrate Y_X/S_, and as a consequence an increase in biomass yield (x) was observed. This, however, had a negative effect in the context of conversion yield of storage lipids and resulted in a reduction in values of all relevant kinetic parameters for the synthesis of SCO. Volumetric rate of storage lipids production (q_Lv_) and specific rate of storage lipid production (q_L_) indicated that the amount of synthesized lipids per unit of time reached a value similar to that in MG7 medium (respectively q_Lv_: 0.019 and 0.015 g/L/h and qL: 0.0017 and 0.0018 g/g_d.w._/h). Therefore, ex novo production of lipids should be carried out in cultures shorter than the de novo route, or the molar ratio of the carbon to nitrogen source should be increased. Storage lipids synthesis by ex novo pathway is commonly claimed to be independent of the nitrogen content in the culture medium but on the other hand some limits in medium compositions are often applied for maximal oil yield. It is worth mentioning what is generally stressed, that when growth is carried out on hydrophobic substrates by ex novo accumulation, the microbial lipid produced contains lower quantities of triacylglycerols compared with growth on sugar-based substrates (de novo synthesis) [5].

Gao et al. [18] cultured *Y. lipolytica* CICC 31596 in a medium containing glucose 2.5 g/L without pH regulation at 28 °C, as in this study. The kinetic parameters were lower than for *Y. lipolytica* KKP 379 in relation to biomass yield and maximum concentration of lipids produced (0.802 g/L) because of the lowest substrate concentration. However, it turned out Y_X/S_ and Y_L/S_, (respectively 0.933 g_d.w._/g and 0.32 g/g of substrate) reached higher values compared to the analogous parameters calculated for yeast culture in MG7 medium, what could be due to strain-dependent effectiveness of SCO synthesis.

The cellular lipids obtained by Bialy et al. [19] in batch flask *Y. lipolytica* culture in medium containing glucose (30 g/L) amounted to significantly less linoleic acid (10.76%), oleic acid (47.28%), and palmitic acid (1.17%) and much more myristic acid (15.37%) than the oil obtained in batch bioreactor culture in this study. Najjar et al. [17] performed cultivation of yeast strain *Y. lipolytica* CBS 7504 in flasks containing 5 cm^3^ YP medium consisting of 1% yeast extract, 2% peptone, and 1% olive oil and observed the presence of a similar amount of palmitic and stearic acids in cellular lipids than those obtained in MO5 medium. At the same time, the lowest concentration of linoleic acid and the absence of palmitoleic acid differentiate the results. Research by Bialy et al. [19] and Najjar et al. [17] were conducted in shaken cultures, on a smaller scale than our research. Papanikolaou et al. [20] observed 7–18% of oleic acid and 56–68% of stearic acid in lipids produced by *Y. lipolytica* ACA-DC 5010 during batch bioreactor culture in nitrogen limited medium with high concentrations of glucose (150 g/L) and stearin (up to 20% wt/wt). Summarizing, it can be concluded that yeast *Y. lipolytica* accumulates storage lipids mainly in the form of triacylglycerols, but as it was proved, the detailed composition of microbial oil depends on the strain, culture conditions, and source of carbon used in the medium.

An increase in oleic acid content from initial substrate, which was rapeseed oil, to cellular lipids was also observed in shaken cultures of *Y. lipolytica* YB 423-12. At the same time, the decrease in palmitic acid concentration occurred [21]. Similar decreases of palmitic acid from 25% to less than 15% was observed for *Y. lipolytica* ACA-DC 50109 grown in medium containing 10 g/L stearin and 11 g/L glycerol. At the same time, the content of stearic acid increased from 52% in the initial substrate to 71.5% in microbial oil, and oleic acid content increased from 2% to 7%. Papanikolaou and Aggelis [7] indicated that the synthesis of microbial oil by ex novo mechanisms allows the modification of the composition of storage lipids relative to the substrate, which in the future can be used to produce lipids with the desired composition by the food or chemical industry.

### 4.2. POX and ACL Genes as Markers of the Ex Novo and De Novo Routes for SCO Synthesis

*Y. lipolytica* yeast has the ability to synthesize storage lipids using two different metabolic pathways. The key enzyme in the synthesis of microbial oil by the de novo route is an ATP-dependent citrate lyase encoded by the *ACL* gene. Acl protein catalyzes the synthesis of acetyl-CoA by citrate breakdown in the cytoplasm, and the product of the reaction is a precursor in the further synthesis of fatty acids [5]. Storage lipid synthesis by the ex novo route involves the use of hydrophobic substrates as a carbon source gained in β-oxidation. The enzymes from the group of acyl-coA oxidases catalyze the first reaction in the process. The Aox2 protein encoded by the *POX2* gene is characterized as an oxidase with the specificity towards long-chain fatty acids. Intermediate products of β-oxidation can be redirected to lipid bodies, where they are stored in the form of triacylglycerols [22].

Fatty acids, which are present in acylglycerols of lipids, are claimed to have an inhibitory effect on the FAS complex (fatty acid synthase complex) and ATP-dependent citrate lyase (Acl, key enzyme in the de novo pathway) [5]. However, the level of *ACL* expression presented in this article was comparable for all cultures.

The expression of the *ACL* gene was low throughout the entire culture period, which corresponded with the thesis that in medium with a lipid carbon source the ex novo SCO synthesis occurs. On the other hand, the identified low level of mRNA of the *ACL* gene was not expected when olive oil was used as the carbon substrate. Generally, Acl enzyme is considered to be inhibited by the presence of exogenous long aliphatic chains (e.g., fatty acids) [5,23]. Therefore, the yeast *Y. lipolytica* ACA-DC 50109 cultured in medium with stearin and glycerol or glucose produced some intra-cellular lipids by the de novo route despite the presence of long-chain fatty acids in medium, which was deduced on the basis of extensive studies on the profile of fatty acids in microbial oil [20,24].

## 5. Conclusions

The results presented here conclude that the composition of the microbial oil of *Y. lipolytica* yeast is strictly dependent on the carbon substrate, yeast strain, and culture conditions. The carbon source in the culture medium strongly affected the kinetics and amount of accumulated lipids in yeast cells and determined biochemical processes including activation of different genes. Interestingly, olive oil (lipid carbon substrate) present in the culture medium contributed to the increased expression of the *POX2* gene, while it did not inhibit the level of *ACL* gene transcription characteristics for de novo lipid accumulation and claimed to be inhibited by fats used as the sole carbon and energy source. Despite the significant dependence of the *POX2* gene expression on the presence of lipid carbon source in medium, a similar correlation for the *ACL* gene could not be found. The expression level of the ATP-dependent citrate lyase gene did not differ between cells sampled from the glucose culture medium and the olive oil culture medium. Therefore, the obtained results presented in the paper incline us to a hypothesis that in the presence of lipid carbon substrates, the synthesis of storage lipids occur in parallel with the use of two pathways (de novo and ex novo) and the *ACL* gene is not inhibited at the translation level by fatty acids incorporated into the yeast cells.

It is also worth investigating into other genes that would allow us to identify not only de novo pathway in yeast cells but also the ex novo route.

## Figures and Tables

**Table 1:** Kinetic parameters of storage lipid biosynthesis in a batch bioreactor culture of *Y. lipolytica* in MG7, MO5, and control YPG medium (* for a better comparison of yeast cultures, the values of kinetic parameters in brackets refers to the end of culture in MO5 medium; values without brackets in column for MO5 medium refers to the time of culture in which the highest value of concentration of lipids produced was determined).

Parameter	Unit	Medium		
		MG7	MO5	YPG
Initial concentration of carbon source (S)	g/L	70	50	20
Time* (t)	h	65	23 (64)	65
Biomass yield (x)	g_d.w._/L	8.60	7.09 (10.890)	13.53
Maximum concentration of lipids produced (L_max_)	g/L	0.994	1.468 (1.200)	0.057
Conversion yield of biomass per carbon substrate (Y_X/S_)	g_d.w._/g	0.123	0.142 (0.218)	0.677
Conversion yield of storage lipids per biomass formed (Y_L/X_)	g/g_d.w._	0.116	0.207 (0.110)	0.004
Conversion yield of storage lipids per carbon substrate (Y_L/S_)	g/g	0.014	0.029 (0.024)	0.003
Volumetric rate of storage lipids production (q_Lv_)	g/L/h	0.015	0.064 (0.019)	0.001
Specific rate of storage lipid production (q_L_)	g/g_d.w._/h	0.0018	0.0090 (0.0017)	0.00006
